# Evolutionary insights from comparative transcriptome and transcriptome-wide coalescence analyses in *Tetrastigma hemsleyanum*

**DOI:** 10.1186/s12870-018-1429-8

**Published:** 2018-09-24

**Authors:** Yihan Wang, Weimei Jiang, Wenqing Ye, Chengxin Fu, Matthew A Gitzendanner, Pamela S Soltis, Douglas E Soltis, Yingxiong Qiu

**Affiliations:** 1 Key Laboratory of Conservation Biology for Endangered Wildlife of the Ministry of Education, and College of Life Sciences, Zhejiang University, Hangzhou, 310058 China; 2 Department of Biology, University of Florida, Gainesville, FL 32611 USA; 3 Florida Museum of Natural History, University of Florida, Gainesville, FL 32611 USA; 4 College of Life Sciences, Henan Agricultural University, Zhengzhou, 450002 China

**Keywords:** coalescent-based analyses, demographic history, gene flow, *K*_*a*_/*K*_*s*_, single-copy nuclear gene, *Tetrastigma hemsleyanum*, transcriptome

## Abstract

**Background:**

*Tetrastigma hemsleyanum* is of great medicinal importance and used as a model system to address the evolutionary history of warm-temperate evergreen (WTE) forest biomes in East Asia over Neogene time scales. However, further studies on the neutral and adaptive divergence processes of *T. hemsleyanum* are currently impeded by a lack of genomic resources*.* In this study, we *de novo* assembled and annotated a reference transcriptome for two cpDNA lineages (Central-South-East vs. Southwest) of *T. hemsleyanum*. We further used comparative genomic and multilocus coalescent approaches to investigate the tempo and mode of lineage diversification in *T. hemsleyanum.*

**Results:**

A total of 52,838 and 65,197 unigenes with an N50 of 1,667 and 1,841 bp for Central-South-East (*CSE*) and Southwest (*SW*) lineages, respectively, were recovered, and 6,692 putative orthologs were identified between the two lineages. Estimation of *K*_*a*_/*K*_*s*_ ratios for these orthologs revealed that ten genes had *K*_*a*_/*K*_*s*_ values significantly greater than 0.5 (*P* < 0.05), whereas 2,099 (*K*_*a*_/*K*_*s*_ < 0.5, *P* < 0.05) were inferred to be under purifying selection. Based on three bioinformatic strategies, we identified a total of 1,018 single-copy nuclear genes (SCNGs) from the orthologs. We successfully designed eight nuclear gene primer pairs with high intraspecific variation (e.g. *h*_T_ = 0.923, *π*_T_ = 1.68×10^-3^), when surveyed across a subset of *T. hemsleyanum* individuals. Concordant with the previous cpDNA data, the haplotype networks constructed for most nuclear gene loci clearly identified the two lineages. A multilocus coalescence analysis suggested that the separation between the two lineages appears to have occurred during the mid-Pliocene. Despite their ancient divergence, both lineages experienced expansion at rather localized scales and have continued to exchange genes at a low rate.

**Conclusions:**

This study demonstrated the utility of transcriptome sequencing as a basis for SCNG development in non-model species and the advantages of integrating multiple nuclear loci for phylogeographic and phylogenetic studies.

**Electronic supplementary material:**

The online version of this article (10.1186/s12870-018-1429-8) contains supplementary material, which is available to authorized users.

## Background

*Tetrastigma hemsleyanum* Diels et Gilg (Vitaceae) is a diploid, perennial herb, with red berries dispersed by birds, bats and mammals, and is distinguished as the sole herbaceous climber of a genus that otherwise comprises only woody lianas [1–4]. This species is widely distributed throughout subtropical China, but also occurs rarely farther south on Hainan and Taiwan [5, 6]. The tubers of *T*. *hemsleyanum* have long been used in Chinese folk medicines to treat hepatitis, fever, pneumonia, rheumatism, and sore throat [7], along with other uses that exploit its anti-tumor effects [8, 9]. Thus, the species has been the subject of considerable phytochemical and pharmacological studies (e.g. [10]). In addition, since *T*. *hemsleyanum* is a typical component of warm-temperate evergreen (WTE) forest habitats in subtropical China, it has emerged as an excellent model species to address the evolutionary history of WTE forest biomes in eastern Asia over Neogene time scales.

Our previous phylogeographic survey using chloroplast (cp) DNA sequences showed that the modern range of *T. hemsleyanum* comprises two major cpDNA lineages, Southwest (*SW*) and Central-South-East (*CSE*) China. Moreover, our recent studies revealed that phenotypic traits, e.g. leaf size, tuber size, and the effect of phytochemical compounds, differ greatly between the two lineages [11]. The two major lineages likely diverged through climate/the uplift of QTP-induced vicariance of an ancestral southern range during the early Pliocene [6]. Nevertheless, given that this climber of the WTE forest is primarily dispersed by frugivorous birds [12, 13] and shows a nearly continuous range at present, we therefore presume that adaptive divergence processes may play an additional role in maintaining the separation of two cpDNA lineages of *T. hemsleyanum* inhabiting different floristic regions [6]. In fact, it has been recognized that Quaternary climatic changes have affected the demographic and adaptive processes in many species, especially in boreal and temperate regions undergoing glacial cycles [14–16]. However, a paucity of genetic resources such as genomic and transcriptomic sequences has made further studies on the neutral and adaptive divergence processes of *T. hemsleyanum* and other non-model species a challenging task.

Recent advances in next-generation sequencing (NGS) and bioinformatic tools have generated genome-scale information for both model and non-model species. Access to these massive sequence data provides researchers with exciting opportunities to make large-scale comparisons at genomic, exomic, or transcriptomic levels [17–19] and develop hundreds of informative, taxon-specific loci from nuclear genomes [20]. Massively parallel sequencing of RNA (RNA-Seq), in particular, has emerged as a powerful and cost-efficient tool to obtain the expressed sequences of the genome in non-model species when other genomic resources, such as a sequenced genome, are not yet developed. Through this approach, data are obtained on nucleotide variation as well as transcriptome characteristics and gene expression levels, substantially improving phylogeographic studies of population history, demography, genetic structure, and adaptive evolution [21]. For example, genome-wide scans and comparative transcriptome analysis not only provide the opportunity to estimate transcriptome-wide divergence and identify loci under selection [22, 23], but also enable the mining of polymorphic molecular markers such as single-copy nuclear genes (SCNG) and microsatellites (SSR) for population genetic and phylogeographic studies [24, 25]. With the increasing abundance of sequence data from across the genome, researchers can use multilocus coalescent methods to effectively estimate demographic parameters on a species tree to help disentangle the historical context of divergence and (incipient) speciation.

In this study, using the Illumina HiSeq3000 platform, we obtained RNA sequence data for two individual samples of *T. hemsleyanum* that correspond to the *CSE* and *SW* lineages [6], respectively. Our aims were to: 1) characterize the transcriptomes of the two lineages of *T. hemsleyanum*; 2) perform pairwise comparisons of the putatively orthologous sequences from these lineages to identify candidate genes under selection that might be involved in local adaptation and maintenance of lineage boundaries; 3) identify large numbers of putative SCNG and expressed sequence tag–simple sequence repeat (EST-SSR) markers and validate the polymorphism of each SCNG locus in a subset of *T. hemsleyanum* individuals; and 4) analyze SCNG sequence data with coalescent-based methods to estimate the history and timing of lineage divergence and the amount of post-divergence gene flow between the two lineages of *T. hemsleyanum*.

## Results

### De novo assembly and functional annotation of unigenes

After filtering and evaluating the raw reads, a total of 47,880,882 and 122,587,340 clean reads were generated by Illumina Sequencing from the cDNA libraries of the *CSE* and *SW* lineages, respectively (Table 1). The GC percentage and Q20 percentage (percentage of sequences with sequencing error rate lower than 1%) were 45.13% and 97.98%, respectively, for the *CSE* lineage and 46.10% and 98.15%, respectively, for the *SW* lineage (Table 1). Through *de novo* assembly, we obtained 101,421 contigs with a mean length of 413 bp and an N50 value of 1,001 bp for the *CSE* lineage, and 138,294 contigs with a mean length of 377 bp and an N50 value of 926 bp for the *SW* lineage (Table 1). For *CSE* lineage sequences, the contigs were assembled into 52,838 unigenes with an average length of 1,034 bp and an N50 value of 1,667 bp, while for the *SW* lineage, 65,197 unigenes were obtained with a mean length of 1,095 bp and an N50 value of 1,841 bp (Table 1). Detailed information on *de novo* assembly is summarized in Table 1, and the length distributions of the contigs and unigenes of the two lineages are shown in Additional file 1: Figure S1.

**Table 1:** Summary statistics for the transcriptomes of *CSE* and *SW* lineages of *Tetrastigma hemsleyanum*.

	CSE lineage	SW lineage
Total number of clean reads	47,880,822	122,587,340
Total length of clean reads (bp)	4,309,273,980	12,258,734,000
Total numbers of contigs	101,421	138,294
N50 value of contigs	1001	926
Mean length of contigs (bp)	413	377
Q20 percentage (%)	97.98%	98.51%
GC percentage (%)	45.13%	46.10%
Total numbers of unigenes	52,838	65,197
N50 value of unigenes (bp)	1667	1841
Mean length of unigenes (bp)	1034	1095

Notes: Q20 percentage denotes the percentage of sequences with sequencing error rate lower than 1%; N50 means that the contig size such that 50% of the entire assembly is contained in contigs equal to or longer than this value, *bp* = base pair.

The sequence similarity searches found that 48,697 (92.16%) non-redundant unigene sequences from the *CSE* lineage and 58,622 (89.92%) from the *SW* lineage had at least one significant match to the priority-ordered protein databases (i.e. Nr, Nt, Swiss-Prot, KEGG, COG) (Table 2). For both lineages, a blastx top-hit species distribution of gene annotations showed the highest similarity to the closely related *Vitis vinifera* [35,546 of 38,451 hits in the *CSE* lineage (92.44%) vs. 38,689 of 43,172 hits in the *SW* lineage (89.62%)], followed by *Amygdalus persica* (0.98% vs 1.18%).

**Table 2:** Annotation results of assembled unigenes from *CSE* and *SW* lineages.

	Functional annotations							CDS annotations		
	Nr	Nt	Swiss-Prot	KEGG	COG	GO	All	Homolog	estscan	All
*CSE* lineage										
Number (N)	38,451	47,387	24,383	22,887	14,520	28,064	48,697	49,915	1056	50,971
N/All annotated (%)	78.96	97.31	50.07	47.00	29.82	57.63	100			
N/All-unigene (%)	72.77	89.68	46.15	43.32	27.48	53.11	92.16	94.47	2.00	96.47
*SW* lineage										
Number (N)	43,172	56,208	27,246	25,659	16,932	30,177	58,622	54,373	1449	55,822
N/All annotated (%)	73.64	95.88	46.48	43.77	28.88	51.48	100			
N/All-unigene (%)	66.22	86.21	41.79	39.36	25.97	46.29	89.92	83.40	2.22	85.62

Based on the Nr annotations, 28,064 (53.11%) unigenes for the *CSE* lineage and 30,177 (46.29%) unigenes for the *SW* lineage were assigned to at least one GO term annotation under three GO categories: biological process (*CSE*: 21,742, 41.15%; *SW*: 23,371, 35.85%), cellular component (*CSE*: 22,123, 41.87%; *SW*: 23,473, 36.00%) and molecular function (*CSE*: 21,296, 40.30%; *SW*: 23166, 35.53%) (Additional file 2: Figure S2). The GO category distributions of the unigenes for both lineages were highly similar. For each lineage, the two mostly highly represented level-2 categories of ‘biological process’ were ‘cellular process’ (*CSE*: 17,548, 62.5%; *SW*: 18,658, 61.8%) and ‘metabolic process’ (*CSE*: 17,411, 62.0%; *SW*: 18,592, 61.61%); of ‘metabolic process’, ‘cell’ (*CSE*: 20,754, 73.95%; *SW*: 22,093, 73.21%) and ‘cell part’ (*CSE*: 20,753, 73.95%; *SW*: 22,092, 73.21%); and of ‘molecular function’, ‘catalytic activity’ (*CSE*:13,703, 48.83%; *SW*: 14,599, 48.38%) and ‘binding’ (*CSE*: 14,006, 49.91%; *SW*: 15,749, 52.19%) Additional file 2: (Figure S2).

### Prediction of protein-coding sequence (CDS) regions

According to the blast search results against various protein databases, we identified and extracted the CDS regions of 49,915 unigenes in the *CSE* lineage and 54,373 in the *SW* lineage, which were then translated into amino sequences with the standard codon table. The respective size distributions of these nucleotide sequences in each lineage (*CSE*/*SW*) were as follows: 100–1000 bp (27,219/30,342), 1,000–2,000 bp (7,762/8,660), and > 2000 bp (2,535/3,072) (see Additional file 1: Figure S1). Beyond that, 1,053 unigene sequences in the *CSE* lineage and 1,499 in the *SW* lineage with CDS were obtained using the estscan software prediction. The shortest estscan CDS were at least 200 bp, and the most abundant size class was 200–500 bp for both the *CSE* and *SW* lineages (831 vs. 1,182, respectively), constituting 78.70% and 81.57% of the total unigenes, respectively (Additional file 1: Figure S1).

### Identification of orthologous contigs and comparison of substitution rates between lineages

Based on the predicted protein sequences, we identified 17,738 putative orthologous groups between the two lineages (*CSE*/*SW*) using orthomcl. The one-to-one, reciprocal best method of elucidating orthologous proteins generated 9,924 putative orthologous pairs (i.e. two sequences from the different lineages have higher blast scores with each other than with any other sequences in the other genome). After filtering gene pairs annotated with different protein products in the Swiss-Prot database, 6,692 pairs of putative orthologs were finally identified and used in the downstream analyses. Of these, 1,786 pairs had only synonymous or non-synonymous substitutions, and 4,906 pairs had both types of substitutions, for which the *K*_*a*_/*K*_*s*_ ratio were calculated. The mean values of *K*_*a*_, *K*_*s*_, and the *K*_*a*_/*K*_*s*_ ratio of all orthologous pairs were 0.011 ± 0.034, 0.051 ± 0.259, and 0.326 ± 0.438, respectively. Of the 4,906 pairs of putative orthologs, only 329 had a *K*_*a*_/*K*_*s*_ ratio >1, and 840 had a *K*_*a*_/*K*_*s*_ ratio between 0.5 and 1. Of the 1,169 putative orthologous pairs with a *K*_*a*_/*K*_*s*_ value > 0.5, none had a *K*_*a*_/*K*_*s*_ ratio significantly greater than 1, and only ten had a ratio significantly greater than 0.5 (see corresponding *K*_*a*_/*K*_*s*_ ratios and gene functions in Table 3). By contrast, over half of the putative orthologous pairs (3,737, 55.84%) showed a *K*_*a*_/*K*_*s*_ ratio < 0.5, of which 2,099 pairs had ratios significantly < 0.5 (*P* < 0.05).

**Table 3:** List of candidate orthologs potentially under positive selection in the transcriptomes of *CSE* and *SW* lineages

Gene ID		*K*_*a*_/*K*_*s*_ value	*P*-value (Fisher)	Descriptions
CSE lineage	SW lineage			
Unigene21284	CL1668_Contig1	4.13	0.002	Type 2 ribosome-inactivating protein Nigrin l precursor
CL1485_Contig2	CL876_Contig13	1.94	0.017	TMV resistance protein N-like
Unigene9215	CL4926_Contig1	0.55	0.001	GDSL esterase/lipase EXL3
CL7288_Contig1	CL7933_Contig2	0.5	0.006	IAA-amino acid hydrolase ILR1-like 4
CL2482_Contig1	CL3284_Contig1	0.51	0.015	Probable disease resistance protein RDL6/RF9-like
Unigene1115	Unigene8463	0.53	0.022	Proline-rich receptor-like protein kinase PERK9
Unigene22333	CL1306_Contig1	0.52	0.029	Probable glycosyltransferase At3g07620-like
CL291_Contig2	CL4212_Contig1	0.59	0.036	Armadillo repeat-containing protein 7
CL6365_Contig1	Unigene18910	0.52	0.046	Glutamyl-tRNA(Gln) amidotransferase subunit A
CL1077_Contig1	Unigene25101	0.52	0.049	Probable disease resistance protein At1g15890

Taking a more appropriate threshold of 0.5 for the *K*_*a*_/*K*_*s*_ ratio as an indicator of positive selection [26], ten putative orthologous pairs might have experienced relaxed purifying selection and/or unfixed mutation, whereas 2,099 were inferred to be under purifying selection. We clustered the above 2,109 putative orthologous pairs into three main GO categories: biological process (1,556, 74.13%), cellular component (1,572, 74.94%), and molecular function (1,459, 69.51%). Within the biological process category, the term ‘cellular process’ (1,269, 61.74%) and ‘metabolic process’ (1,249, 59.50%) were the most dominant. Within the cellular component category, ‘cell’ (1,503, 71.61%) and ‘cell part’ (1,502, 71.56%) represented the major subcategories. Within the molecular function category, the main functional subcategories are ‘binding’ (947, 45.12%) and ‘catalytic activity’ (911, 43.40%) (Additional file 2: Figure S2).

### Identification and characterization of EST-SSRs

A total of 11,006 and 15,531 EST-SSRs were identified from 9,054 (*CSE*) and 12,486 (*SW*) unigenes, accounting for 17.14% and 19.15% of the total unigenes in *CSE* lineage (52,838) and *SW* lineage (65,197), respectively. From the SSR-containing unigenes, 1,590 (*CSE*) and 2,402 (*SW*) had more than two EST–SSR loci. The most abundant repeat types in the *CSE* lineage were trinucleotide (3,679, 33.43%), followed by dinucleotide (3,289, 29.88%), and mononucleotide (2,929, 26.61%). These three SSR repeat types were also found to be the most highly represented in the *SW* lineage (mononucleotide: 4,904, 31.58%; dinucleotide: 4,500, 28.97%; trinucleotide: 4,649, 29.92%) (Additional file 3: Figure S3). The dominant EST-SSRs identified here were A/T (*CSE*: 2,924, 29.54%; *SW*: 4,819, 34.29%), AG/CT (*CSE*: 2,381, 24.06%; *SW*: 3,236, 23.03%), and AAG/CTT (*CSE*: 1,022, 10.33%; *SW*: 1,316, 9.36%) (Additional file 4: Figure S4). Very few CG/CG repeats (*CSE*: 2, 0.02%; *SW*: 2, 0.01%) were identified in the two databases (Additional file 4: Figure S4).

To maximize the universal applicability of markers, we also searched for SSRs in the 6,692 pairs of putative orthologs, and found 286 SSRs distributed among 200 pairs of orthologs (Table 4). Of the 286 SSRs loci, 78 exhibited variation in the number of specific repeat units between the two lineages and should be the best choices for future population genetic studies of this genus.

**Table 4:** Test results of polymorphism and neutrality for each SCNG screened in representative individuals of *T. hemsleyanum*

Locus ID	*S*	*N*	Haplotype diversity (*h*_T_)	Nucleotide diversity (*π*_T_)	Test of neutrality					
					Tajima’s *D*	*P*	Fu and Li’s *D*	*P*	Fu and Li’s *F*	*P*
Th-41	22	19	0.960	0.00944	-0.586	*P* > 0.01	-0.457	*P* > 0.01	-0.590	*P* > 0.01
ThR-3	41	32	0.991	0.01185	-0.684	*P* > 0.10	0.932	*P* > 0.10	0.451	*P* > 0.10
ThR-6	41	31	0.989	0.01527	-0.014	*P* > 0.10	0.980	*P* > 0.10	0.759	*P* > 0.10
ThR-7	40	15	0.923	0.01682	1.049	*P* > 0.10	-0.183	*P* > 0.10	0.275	*P* > 0.10
ThR-11	23	25	0.979	0.00798	-1.228	*P* > 0.10	-0.548	*P* > 0.10	-0.910	*P* > 0.10
ThR-28	31	21	0.960	0.00946	-0.819	*P* > 0.10	-0.697	*P* > 0.10	-0.870	*P* > 0.10
ThR-31	24	19	0.919	0.01380	-0.172	*P* > 0.10	-0.754	*P* > 0.10	-0.663	*P* > 0.10
ThR-34	25	27	0.981	0.01027	-0.559	*P* > 0.10	0.871	*P* > 0.10	0.458	*P* > 0.10

Number of polymorphic sites, *N* Numbers of haplotypes.

### Mining of SCNGs and multilocus coalescence analyses

**Fig. 1:**
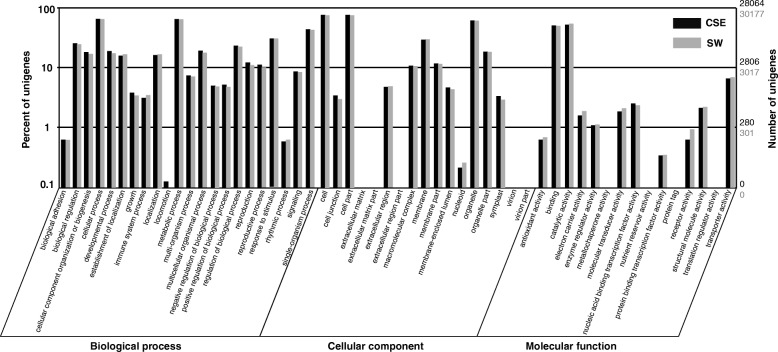
Distribution of gene ontology (GO) term categories in the transcriptomes of *CSE* and *SW* lineages. They-axis indicates the percent of unigenes (left y-axis), and the number of unigenes (right y-axis) per (sub-) category. Black bar, *CSE* lineage; grey bar, *SW* lineage.

A total of 353 and 167 genes from transcriptome data of both the *CSE* and *SW* lineages were found to have hits against the SCNG sets of APVO and Vitaceae species, respectively. Of the 520 putative SCNGs, 34 were shared between the results of the above two approaches. In addition, 730 putative SCNGs were identified by markerminer, of which 19 were classified as ‘strictly single-copy’, and 711 as ‘mostly single-copy’ (Fig. 1, Additional file 5: Table S2). Among the 730 SCNGs identified by markerminer, 198 were detected by the first two approaches (Fig. 1). The final set consisted of 1,018 putative SCNGs (Fig. 1, Additional file 5: Table S2). These genes were most likely to be SCNGs in both lineages.

Pairwise alignments of all the 1,018 SCNGs demonstrated that a large proportion of loci yielded medium to high levels of sequence divergence between the two lineages: approximately one third of those loci (346, 34%) were estimated to have greater than 1% (identity < 99%) sequence divergence, and nearly half (451, 44.3%) showed 0.5%–1% divergence for the inter-lineage comparisons (Additional file 6: Figure S5 and Additional file 5: Table S2). Notably, 417 of these putative SCNGs showed significant signals of purifying selection (*K*_*a*_/*K*_*s*_ < 0.5, *P* < 0.05) (see blue dots in Additional file 6: Figure S5). To test the utility of the 1,018 putative SCNGs for phylogeographic and phylogenetic studies in *T. hemsleyanum*, 50 candidate loci were randomly selected for primer design, and 23 yielded a single band at the expected size range. Of these 23 primer pairs, 12 were confirmed to be polymorphic in the initial screening with the 15 individuals from the five populations by Sanger sequencing (primer sequences and amplification results presented in Additional file 5: Table S1 and Additional file 7: Figure S6). However, four loci (ThR-4, ThR-5, ThR-8, ThR-10 ) were found to be less informative, and were excluded from further analyses.

The eight candidate loci (Th-41, ThR-3, ThR-6, ThR-7, ThR-11, ThR-28, ThR-31, and ThR-34) that showed relatively high intraspecific variation were finally chosen to genotype all individuals listed in Additional file 5: Table S3. The aligned sequences of each of the eight amplified loci for the 30 samples from the two lineages ranged from 463 to 895 bp (see Additional file 5: Table S3 for accession numbers). We observed a total of 247 polymorphic sites, with the number for each locus varying from 20 to 41. For each locus, the number of haplotypes identified ranged from 19 to 32, with a mean of 24 haplotypes per locus. The levels of haplotype (*h*_T_) and nucleotide (*π*_T_) diversity were consistently high, with each marker varying from 0.909 to 0.993, and from 7.32 × 10^-3^ and 16.01 × 10^-3^, respectively (Table 4). At the lineage level, *SW* (*h* = 0.815; *π*= 5.32 × 10^-3^) exhibited, in general, lower levels of diversity than *CSE* (*h* = 0.992; *π*= 10.99 × 10^-3^). Tests of neutrality showed no significant departure from the neutral model for each locus (Table 4), so all the eight loci were retained for subsequent coalescence analyses. Most nuclear networks supported the split of the two lineages except for two loci (i.e. ThR11, ThR34), which were unresolved and therefore not conflicting (Additional file 8: Figure S7).

**Fig. 2:**
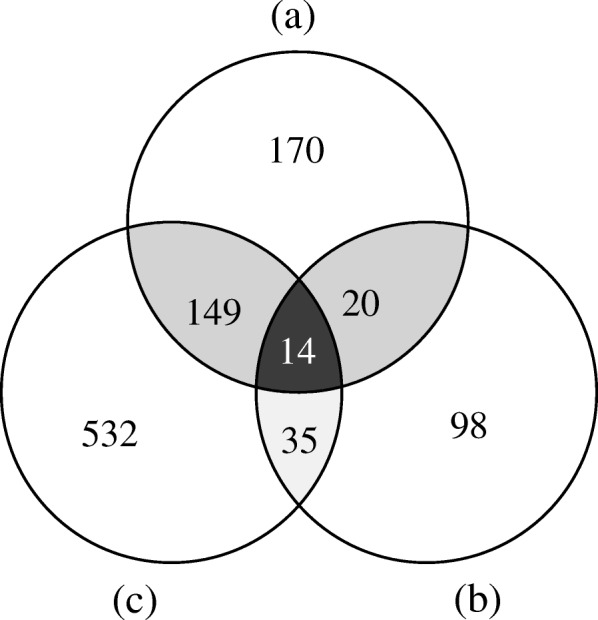
Each of the circles represents the number of SCNG loci homologous to the gene datasets of (**a**) APVO or (**b**) Vitaceae species, or detected by (**c**) the software markerminer. The grey and black overlapping area of the three circles correspond to the number of SCNGs shared between the results of the two, and three approaches, respectively.

For the ima analysis, since cpDNA genealogical patterns [6] were mostly congruent with those retrieved from the nuclear loci, we employed the largest non-recombining blocks of the eight SCNG loci together with the previously obtained cpDNA sequences [6]. The maximum-likelihood estimates (MLEs) and the 90% highest probability density (HPD) intervals of the six ima-derived parameters are summarized in Table 5, and their marginal posterior probability (MPP) distributions are illustrated in Fig. 2. Based on the geometric average mutation rate calculated (*V* = 5.04 × 10^-7^ mutations per locus yr^-1^), these parameter estimates were converted to absolute values of years or individuals. We estimated the time of the split between the *SW* and *CSE* lineages at about 2,964,251 yr bp, with a 90% HPD interval ranging from 1,592,603 to 4,688,583 yr bp. (Table 5, Fig. 2). The current effective population size (*N*_e_) of each descendant lineage (*N*_SW_: 6.67 × 10^5^; *N*_CSE_: 8.45 × 10^5^) was estimated to be much larger than that of the ancestral population (*N*_A_ = 2.7× 10^5^). Peak posterior estimates of post-divergence migrations from the *CSE* to *SW* lineage (*m*_CSE_-_SW_ = 0.048), and vice versa (*m*_SW_-_CSE_ = 0.073), were low (Table 5, Fig.2), corresponding to population migration rate estimates (2*Nm*= *Θm*/2) of 0.204 and 0.245 migrants per generation, respectively.

**Table 5:** Maximum-likelihood estimates (MLE) and 90% highest posterior density (HPD) intervals of demographic parameters of *T. hemsleyanum* based on ima multi-locus analyses.

Estimates	*Θ*_CSE_	*Θ*_SW_	*Θ*_A_	*m*_cse-sw_	*m*_sw-cse_	*t*	*N*_CSE_	*N*_SW_	*N*_A_	2N_CSE_M_CSE-SW_	2N_SW_M_SW-CSE_	*T* (years BP)
MLE	8.518	6.718	2.728	0.048	0.073	1.494	845282	666590	270737	0.201	0.232	2,964,251
HPD90HPD_LO_	4.963	1.888	1.188	0.000	0.000	0.803	492425	187295	117835	0.000	0.000	1,592,630
HPD90HPD_Hi_	12.69	12.96	4.412	0.133	0.226	2.362	1258971	1286259	437849	0.562	0.738	4,688,583

*Θ*_CSE_, *Θ*_SW_, *Θ*_A_ represent the scaled effective population sizes (*N*_e_) of CSE lineage, SW lineage of *T. hemsleyanum*, and the ancestral population, respectively. *m*_cse-sw_ and *m*_sw-cse_ refer to the scaled migration rates forward in time from CSE to SW lineage and vice versa. *t* is the time since ancestral population splitting in mutational units. 2N_CSE_M_CSE-SW_ and 2N_SW_M_SW-CSE_ are the effective migration rates (number of migrants per generation). All estimates include the per gene mutation rate *V* (geometric mean of the mutation rates of all the loci). Parameters in the first six columns are scaled by the mutation rate, while the rest are scaled by years or individuals.

## Discussion

### Assembly and annotation

We sequenced transcriptomes of geographically separated and genetically differentiated lineages (*CSE* vs. *SW*) of the perennial herb *T. hemsleyanum*. Our results are similar to the transcriptome assembly outcome of close relatives of *T. hemsleyanum* in Vitaceae using similar technologies (number of unigenes: 70,977–154,609; N50: 1,098–1,566; [27]). However, the number of protein-coding genes assembled for each lineage of *T. hemsleyanum* (*CSE*: 49,915 genes; *SW*: 54,373) is higher than that obtained in the *Vitis vinifera* genome (30,434 genes; [28]), indicating that our sequencing and assembly captured a considerable fraction of *T. hemsleyanum* protein-coding genes but that our assembly includes multiple unigenes that correspond to a single gene. Moreover, the tripartite nature of the *Vitis* genome (and of other Vitaceae as well as the common ancestor of all core eudicots) means that, in general, there are three copies of each gene relative to a gene from *Arabidopsis thaliana*, which has undergone extensive reduction in gene number relative to *Vitis* (e.g. [28]; *Amborella* Genome Project 2013). Thus, assembly of transcriptomes into unigenes likely produces an overestimate of the true gene copy number as cDNA reads are not all assembled into full-length genes.

**Fig. 3:**
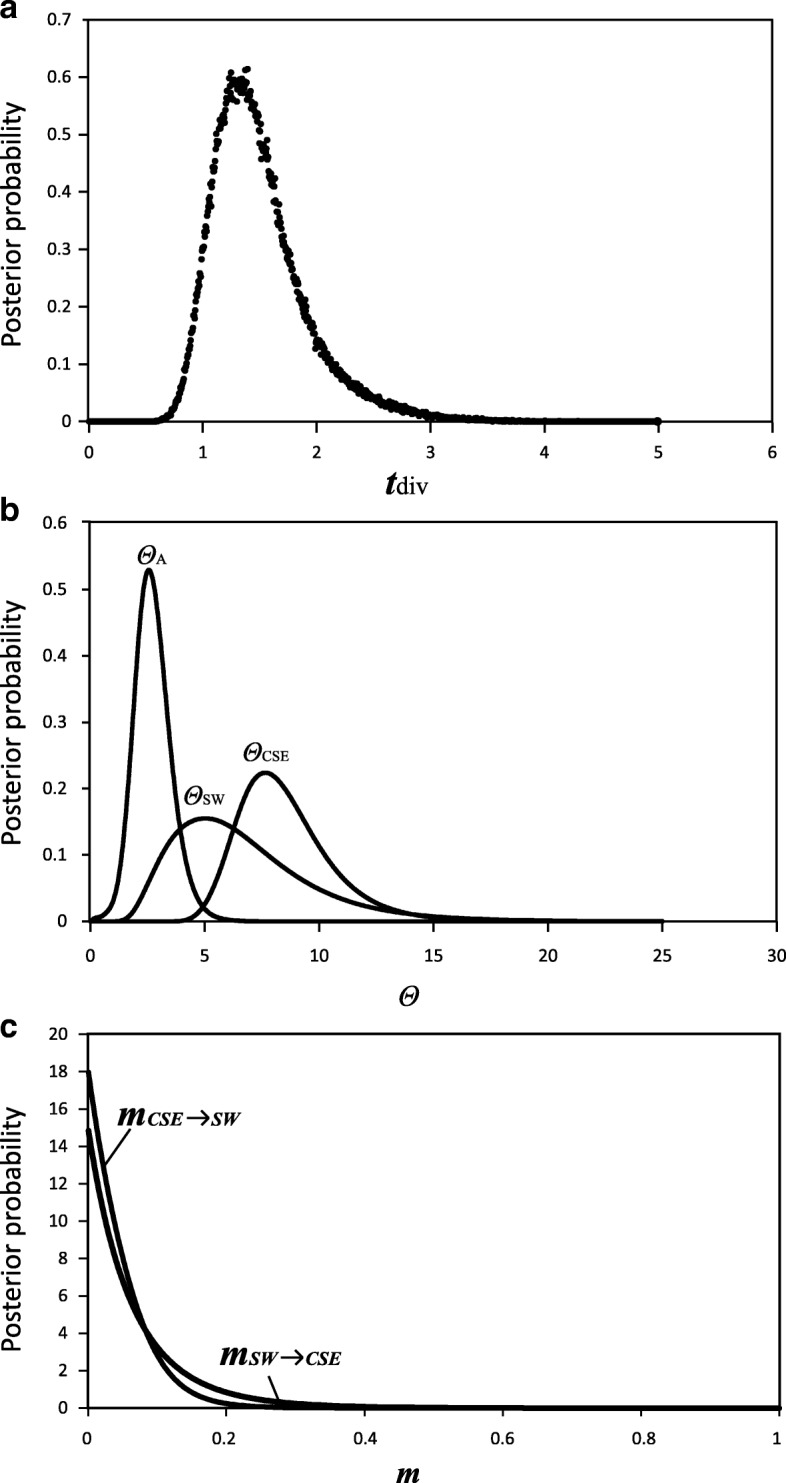
Marginal posterior probability (MPP) distributions of ima model parameters between the Central-south-east (*CSE*) and Southwest (*SW*) lineages identified in *T. hemsleyanum*: (**a**) the time (*t*) since ancestral population splitting in mutational units (**b**) the scaled effective population sizes of both lineages (*Θ*_CSE_, *Θ*_SW_), and the ancestral population (*Θ*_A_) (**c**) the scaled migration rates from *CSE* to *SW l*ineage (*m*_CSE-SW_), and vice versa (*m*_SW-CSE_).

A very high proportion of assembled unigenes matched blast searches to the known proteins in public databases (Nr, Swiss-Prot, KEGG, and COG) (*CSE*: 92.16%; *SW*: 89.92%) (Table 2). Functional annotations of these unigenes were highly similar between the two lineages (Fig. 3), in terms of both the types and relative frequencies of GO categories expressed, suggesting their overall similarity in gene expression profiles. A substantial portion of unigenes with blastx hits to Nr databases shared the highest sequence similarity with the closely related model species *V. vinifera*, which accounted for 92.44% and 89.62% in the *CSE* and *SW* lineages, repectively, with a very number hitting other plant groups. Only a small fraction of unigenes from both the *CSE* (7.84%) and *SW* (10.38%) lineages were not annotated or had no blast matches to protein databases. As expected, these sequences had a much smaller average sequence length (*CSE*: 342 bp; *SW*: 316 bp) and thus were less likely to obtain significant blastx matches [29, 30]. Nevertheless, some of them may also represent novel proteins unique to *T. hemsleyanum*, fast-evolving genes, or untranslated regions (UTRs) [23, 31].

### Nucleotide substitution effect on CDS of the two lineages

To describe genome-wide levels of coding sequence evolution and to estimate the effects of selection on lineage divergence, we calculated the ratio of *K*_*a*_/*K*_*s*_ for orthologs of the *CSE* and *SW* lineages. This ratio has frequently been used as an indicator of frequency and mode of selection under which a protein-coding gene is evolving [32, 33]. Of the 4,906 orthologs shared between lineages that permit the calculation of *K*_*a*_ and *K*_*s*_, only two gene pairs exhibited *K*_*a*_/*K*_*s*_ ratios significantly >1, and an additional eight putative orthologs were above the less stringent threshold of 0.5 (Table 3). These genes were involved in several biological functions (e.g. disease resistance, metabolic process; Table 3) and may constitute candidates that are under the effect of positive selection or relaxed purifying selection and that are thus potentially associated with lineage divergence. Nearly half of the remaining orthologous pairs (2,099, 42.9%) had *K*_*a*_/*K*_*s*_ ratios significantly < 0.5 (*P* < 0.05). Moreover, the average *K*_*a*_/*K*_*s*_ ratio across all pairs is much lower than 1 (0.214), suggesting that purifying selection has a general influence on the evolution of most protein-coding regions of the two *T. hemsleyanum* lineages, as has been observed in other plants [34]. In fact, these genes most under the influence of purifying selection (i.e., with *K*_*a*_/*K*_*s*_ near zero) contained primarily structural or “housekeeping” genes, e.g. tyrosyl-tRNA synthetase, transmembrane protein. As these genes are involved in processes that are crucial for organisms, purifying selection has acted to eliminate deleterious, nonsynonymous mutations [35, 36].

Overall, although we detected only a few genes under positive selection between the two lineages of *T. hemsleyanum*, selection might have played a role in shaping the lineage divergence of this species, given that the *CSE* and *SW* lineages are exposed to the Pacific monsoon (with cold winters and warm/humid summers) and the Indian monsoon (with rainy summers and autumns), respectively [37]. In this study, a peak *K*s distribution between *SW* and *CSE* was observed at 0.051 ± 0.259. This value is higher than that found for two *Primula* species (0.027 ± 0.017; [38]) and falls within a mean *K*s value of 0.03 – 0.10 between congeneric species [39], suggesting that the *CSE* and *SW* lineages may be at an incipient stage of allopatric speciation and may each merit species status (see below).

### SCNG discovery and the estimation of divergence between lineages

In this study, we developed two sets of genetic markers, including nuclear microsatellites (EST-SSR) and single-copy nuclear genes, from 6,692 pairs of orthologous unigenes of the two *T. hemsleyanum* lineages. Compared with untranslated regions (UTRs), EST-SSRs located in protein-coding regions are proved to have higher transferability and equivalent levels of polymorphism [40]. Thus, the targeting of exonic repeat motifs might be the best strategy for developing portable sets of polymorphic EST-SSR markers [41]. The advantages of single-copy nuclear genes for phylogeographic and phylogenetic studies have long been recognized because of their high evolutionary rates and clear avoidance of paralogy [42, 43]. As a result, SCNGs have been shown to provide a valid alternative to nrDNA and chloroplast regions for resolving shallow-scale phylogenetics, intraspecific divergence, and population dynamics [6, 44]. SCNG databases have been reported from model plant species in many clades; these genes are expected to evolve in concordance with species evolution, thus providing invaluable resources for mining SCNGs from transcriptomes of non-model species. In this study, using the curated lists of SCNGs reported in *Arabidopsis*-*Populus*-*Vitis*-*Oryza* (APVO, [45]), Vitaceae [27], and 17 genomes broadly distributed across angiosperm phylogeny ([46]; implemented in markerminer, [20]) as the blast query, we identified 353, 167, and 730 putative SCNGs (1,018 in total) in *T. hemsleyanum*, respectively.

The pairwise overlapping SCNG numbers among the three gene sets were 34, 49 and 163, respectively, and only 14 genes were shared by all databases (Fig. 1). Such a low overlapping proportion of genes indicated that the SCNGs identified in different studies vary substantially with the reference databases employed for blast searches. As a result, we processed multiple queries against our putative orthologs, with SCNG datasets reported across different plant group or phylogenetic frameworks, in order to obtain a more comprehensive candidate list of SCNGs that we can use for further validation for both *T. hemsleyanum* and closely related species. Many of the SCNGs identified here, especially those orthologous to loci from Duarte *et al.* [45] and De Smet *et al.* [46], had general “housekeeping” functions, and are supposed to be conserved across species or even higher taxonomic levels. Nevertheless, based solely on exonic regions of these SCNGs, we estimated that 34% of the loci are > 1% divergent between intraspecific lineages of *T. hemsleyanum*, and over 78% showed levels of divergence > 0.5% (Additional file 6: Figure S5). These SCNG loci may represent more desirable choices for shallow-scale phylogeographic surveys when partial or complete intronic regions with faster evolutionary rates are captured along with their exonic counterparts [47].

### Inference of genetic structure and demography based on multiple nuclear loci

We developed an efficient strategy for mining SCNG markers by comparative transcriptome analysis. By testing eight of the candidate markers, we illustrated the usefulness of these markers in population genetic and phylogeographic studies of *T. hemsleyanum*. These eight loci screened in a subset of individuals of *T. hemsleyanum* were informative and revealed ample polymorphism at the species level (*h*_T_: 0.919–0.991, *π*_T_: 0.008–0.017; Table 4). Concordant with the previous cpDNA data [6], haplotype networks reconstructed for each locus revealed that each lineage region mostly harboured a distinct set of haplotypes, and significant divergences were observed at most of the loci between haplotypes found in Southwest China and those restricted to Central or South-east China (Additional file 8: Figure S7). Thus, the general genealogical structure of haplotypes based on the nuclear genes combined revealed the same phylogeographic pattern as did the cpDNA genealogical structure. However, we believe the lack of geographic structure shown by a few loci (ThR11, ThR28, ThR34) is due to their longer coalescence times. The pattern of genetic structure we observed across cytoplasmic–nuclear data sets suggests long-term lineage/regional population isolation and differentiation over multiple glacial/interglacial cycles with little admixture.

Divergence dates estimated from gene trees, including phylogenies estimated from concatenated sequence data, can lead to overestimates of divergence times because gene divergence necessarily predates speciation [48]. In contrast, methods using multiple unlinked loci in conjunction with a coalescent framework, such as models of ‘isolation with migration’ (IM) implemented herein, have been proposed to provide more biologically realistic estimates because multiple unlinked genetic loci provide independent realizations of divergence history, accounting for mutational and coalescent stochasticity [48, 49]. In the present study, our ima analysis of combined multi-locus data (cpDNA and SCNGs) revealed that the separation between the *SW* and *CSE* lineages most likely occurred during the mid-Pliocene, *c*. 2.96 Ma (90% HPD: 1.59–4.69 Ma) (Fig. 2, Table 5). The estimate of lineage divergence time is more recent than that inferred from the previous fossil-calibrated phylogenetic dating of Vitaceae based on cpDNA sequences (*c*. 5.07 Ma). In fact, the new estimate provided here coincides with a long transition from a warm and stable global climate towards a cooler regime during the Piacenzian (3.60–2.58 Ma) [50, 51]. It seems likely, therefore, that the cooling and aridification at the Pliocene-Pleistocene boundary may have induced an ancient vicariant event, which promoted lineage divergence in *T. hemsleyanum*.

In terms of the demographic history, the previous results inferred from mismatch analyses of cpDNA alone [6] suggest long-term population stability of the *SW* lineage and latitudinal range shifts of the *CSE* lineage. In contrast, the current ima analysis suggested a somewhat larger effective population size (*N*e) in both lineages compared to their ancestral population (Table 3, Table 5). The coalescent analyses using more rapidly evolving nuclear genes can trace more recent demographic events [52, 53]. Therefore, based on multiple nuclear genes, our ima analysis may reflect the recent demographic expansion signal in each lineage. Considering the pronounced geographic structure observed in *T. hemsleyanum* across two genetic data sets (SCNGs and cpDNA), the recent expansion dynamics, even for the *CSE* lineage, in which *N*_CSE_ is larger than ancestral *N*_A_ by 2–3 orders of magnitude, likely occur at localized scales. Our coalescent analyses show that despite their ancient divergence, both lineages have continued to exchange genes at a low rate (*m*_CSE_-_SW_ = 0.048; *m*_SW_-_CSE_ = 0.073). This effective rate of gene exchange between the two lineages corresponds to less than one immigrant per generation (2*Nm* < 0.25). Thus, this climate-induced range expansion at regional scales during inter-/postglacial periods presumably provided recurring opportunities for gene exchange between diverging lineages through secondary contact zones, for example, possibly at the southeastern Yungui Plateau. This hypothesis is also supported by previous ENM results [6]. In addition, a few genes involved in resistance to biotic and abiotic stresses were found to be under positive selection between the two lineages, suggesting that ecological forces may be important in the divergence and potentially in the maintenance of lineage boundaries of *T. hemsleyanum.* Thus, the two lineages might have undergone divergent evolution in physiological and/or life history traits resulting from adaptation to different eco-climatic conditions. We consider this as a viable hypothesis because the *CSE* lineage is exposed to the Pacific monsoon, whereas the SW lineage is affected by the Indian monsoon [54]. Future work that includes codon-level analyses, genome-wide association analyses, studies of genomic patterns of introgression along secondary contact zones, and larger numbers of samples from additional localities will shed more light on the specific loci that are most important in speciation, species integrity, and adaptation [55]. However, in view of their differences in some morphological traits and phytochemical composition, together with their deep genetic divergence at nuclear gene loci and cpDNA regions, the two lineages may represent distinct phylogenetic species, irrespective of whether they are reproductively isolated. The results of future studies may therefore have taxonomic implications as well.

## Conclusions

The results illustrate the utility of transcriptome sequencing as a basis for single-copy nuclear gene development in non-model species. Analyses of the eight SCNGs combined revealed the same two groups as did the cpDNA. Our coalescent analyses of the combined data sets (SCNGs and cpDNA) suggested that *T. hemsleyanum* experienced a dichotomous split at *c.* 2.96 Ma and that, although the two groups have increased in population size, the lineages have remained isolated with limited gene flow. Accordingly, the present study demonstrated that multilocus coalescence analyses can improve estimates of process parameters such as divergence time and population expansion and thus offer a powerful complementary data set to cpDNA markers alone in tracing the evolutionary history of recently diverged lineages or species. In addition, comparative transcriptome analysis offers preliminary support for the hypothesis that ecological forces may be important in the divergence and potentially in the maintenance of lineage boundaries of *T. hemsleyanum*. Overall, our transcriptome analysis provides a solid foundation for future studies of gene expression, natural selection, and speciation in *Tetrastigma*.

## Methods

### Plant materials, total RNA extraction, and Illumina sequencing

Fresh juvenile leaf samples of *T. hemsleyanum* were harvested from the two major chloroplast lineages identified in our previous phylogeographic study [6]. As the *CSE* lineage contained two cpDNA sublineages (South/East and Central) [6], leaf materials of the *CSE* lineage were obtained in July 2014 from one individual in South/East sublineages (Ningbo, Zhejiang Province; 30.35°N, 122.32°E, alt. 162 m) and one individual in Central sublineages (Malipo, Yunnan Province; 23.12°N, 104.84°E, alt. 1700 m), while those of the *SW* lineage were collected in April 2015 from one individual in Guilin Botanical Garden, Guangxi Province (transplanted from Quanzhou, Guangxi Province; 25.08°N, 110.30°E, alt. 170 m). No specific collecting permits required for the collection of plant materials. Leaf tissue samples were frozen in liquid nitrogen and stored at -80°C immediately until total RNA extraction. For each individual, total RNA was extracted using a modified CTAB method. Equal amounts of high-quality RNA from two individuals of the *CSE* lineage were subsequently pooled into a single lineage sample. After assessing RNA quality using an Agilent 2100 Bioanalyzer (Agilent, Santa Clara, CA, USA), the quantified total RNAs were sent to Beijing Genome Institute (BGI, Shenzhen, China) for further processing. The cDNA libraries were constructed using a cDNA Synthesis Kit (Illumina, Inc., San Diego, CA, USA) following the manufacturer’s instructions and evaluated with an Agilent 2100 Bioanalyzer and ABI StepOnePlus real-time PCR system prior to Illumina sequencing. Paired-end sequencing (2 x 90 bp) was then performed using a HiSeq3000 (Illumina, Inc, San Diego, CA, USA). Raw sequence reads were deposited in the NCBI Sequence Read Archive (SRA) with accession numbers SAMN07520791 (*CSE* lineage) and SAMN07502792 (*SW* lineage).

### Sequence cleaning, de novo assembly, and gene annotation

Raw sequence reads were processed using the filter_fq program (BGI, Shenzhen, China) to remove sequencing adaptors, low-quality reads with percentage of unknown nucleotides ‘N’ higher than 5%, and percentage of low-quality bases (quality scores < 10) higher than 20%. Sequences that were retained following these filtering steps were *de novo* assembled using trinityv20131110 [56] with the default parameters. For each lineage sample, the assembled unigenes were further processed by sequence splicing and redundancy removal using tgicl v2.1 [57] to acquire the final non-redundant unigene dataset.

All unigenes were queried against the National Center for Biotechnology Information (NCBI) non-redundant (Nr) protein database, the SwissProt protein database (http://www.expasy.ch/sprot), the Kyoto Encyclopedia of Genes and Genomes (KEGG) pathways database, and the Cluster of Orthologous Groups (COG) database (http://www.ncbi.nlm.nih.gov/COG/) using blastx [58] with an E-value cut-off of 1e^-5^. If the aligning results from different databases were not in accordance with each other, a priority order of Nr, Swiss-Prot, KEGG, and COG was followed. Gene ontology (GO) terms [59] of the unigenes were obtained using blast2go v2.6.0 [60] based on the best blastx hits from the NCBI Nr database with an E-value cut-off of 1e^-5^. The distributions of level-2 GO terms were plotted with functional classification using wego [61].

### Prediction of protein-coding sequence (CDS) regions

The coding region sequences (CDS) of unigenes were predicted according to the blast results against the Nr, Swiss-Prot, KEGG, and COG protein databases in that order (*E*-value < 1e^-5^). Unigenes with hits against the high-priority database were not aligned to those of lower priority. The CDS regions of unigenes that could be aligned to the databases were defined based on blastx results and were translated into peptides using the standard codon table. Unigenes without hits against any of the above four databases were screened by estscan v2.1 [62] to predict CDS region, determine the nucleotide sequence direction (5'–3'), and translate into peptide sequences. Both nucleotide and protein sequences of the unigene coding regions were obtained for further analysis.

### Identification of orthologous contigs between lineage-specific transcriptomes and estimation of substitution rates of putative orthologous pairs

In principle, explicit phylogenetic analysis is the most appropriate method for disentangling orthologous and paralogous genes, but they are computationally expensive to construct for large numbers of genes. Previous studies comparing tree-based analysis and heuristic algorithms indicated that, despite conceptual differences, they produce similar sets of orthologs, especially at short evolutionary distances [63]. As a result, in this study, ortholog detection was conducted by similarity methods. Firstly, the predicted CDS regions of both transcriptomes were used as queries and targets respectively to search against those of the other species using all-versus-all blastp method [58]. The protein-coding sequences with unexpected stop codons in the Blast hit region and/or shorter than 150 bp in length were removed. The best hits of the longest isoforms with *E*-value < 1e^-5^ were retrieved. Orthologous pairs with identity < 60% were excluded and only 1:1 orthologous pairs in both lineages were retained. Next, based on the blast results, Markov clustering was conducted using orthomcl v2.0.9 [64] with default settings. Finally, we compared the pairs of sequences against the Swiss-Prot database (*E*-value < 1e^-5^), and only those gene pairs that mapped unambiguously to the same protein were retained as orthologous genes. The software kaks_calculator v1.2 [65] was employed to estimate nonsynonymous (*K*_*a*_) and synonymous (*K*_*s*_) substitution rates, and *K*_*a*_/*K*_*s*_ ratios of each putative orthologous pair using the YN [66] algorithm.

### Identification of EST-SSRs and mining of SCNGs

A Perl script known as MIcroSAtellite (MISA, http://pgrc.ipkgatersleben.de/misa) [67] was employed to identify and localize the mono-to-hexanuclecotide SSR motifs from each of the two non-redundant unigene datasets and also from the putative orthologous pairs identified between lineages. Primers with a repeat-unit length of at least 16 bp were designed for each SSR-containing sequence using primer premier v6.0 (Premier Biosoft International, Palo Alto, CA, USA). The putative function of SSR-containing sequences was obtained from the results of blastx searches of the Cluster of Orthologous Groups (COG) database (http://www.ncbi.nlm.nih.gov/COG/).

In this study, three methods were used for mining single-copy nuclear genes (SCNGs). The first two approaches employed data from the results of two previous studies that used algorithms to identify putative SCNGs at wider taxonomic scales: [58] the 959 SCNGs shared by four model plants (APVO, *Arabidopsis thaliana*, *Populus trichocarpa*, *Vitis vinifera*, and *Oryza sativa*) from the TAIR10 database [45]; and [59] the 417 SCNGs extracted from the transcriptomes of 15 species of Vitaceae [27]. The protein sequences encoded by the above published SCNGs were then queried against the orthologous genes between the lineages of *T. hemsleyanum* using orthomcl v2.0.9 [64] with default settings. All of the queries with top reciprocal blast hits were considered to be putative SCNGs in *T*. *hemsleyanum*. In a third approach, we used markerminer v1.0 [20] to infer the SCNGs between lineages via the iPlant Collaborative Atmosphere cloud-computing infrastructure (https://www.cyverse.org/; [68]). In this analysis, we chose the proteome of *V. vinifera* from the plaza v2.5 database [69] as a reference to filter the putative orthologous pairs. Then a user-specified SCNG reference [46] implemented in markerminer v1.0, which included 177 ‘strictly SCNGs’ (single-copy in all 17 angiosperm reference genomes) and 2,809 ‘mostly SCNGs’ (with duplicates detected in at least one to as many as three other genomes), was chosen as a final data filter for SCNGs. Putative orthologous pairs between lineages whose transcripts have top reciprocal BLAST hits against the reference proteins were retained and classified as putative SCNGs. The sequence difference for each pair of SCNG (identified by all three approaches) of the two *T. hemsleyanum* lineages was observed by sequence alignments and the calculations of pairwise identities performed in muscle v3.8.31 [70]. To predict the intron-exon boundaries of all SCN loci and approximate the intron size, the reference CDS of *V. vinifera* containing introns were aligned to their respective SCNG alignments of the focal species using mafft v7 [71].

### SCNGs validation and multilocus coalescence analyses

To assess the utility of SCNG markers in phylogeographic inference, we designed primers and tested their amplifications in a subset of *T. hemsleyanum* individuals. Specifically, we selected SCNG loci for primer design at random using primer premier v6.0 (Premier Biosoft International, Palo Alto, CA) and verified whether the following three criteria were met for each locus. The locus: [58] was identified as SCNG by at least two bioinformatic approaches; [59] possessed 20%–60% intron content to increase phylogenetic resolution among recently diverged lineages; and [17] ranged in length from approximately 600 to 1200 bp to facilitate the subsequent PCR and sequencing steps.

We selected 50 loci for primer design following standard primer design guidelines and aimed for exon-anchored primers with a length of 18–24 bp, a GC content of 40–60%, melting temperature (*T*_m_) = 55–62°C, and without repeats, runs, secondary structures such as hairpins, dimers, and cross-dimers. Priming sites containing ambiguous bases due to intraspecific polymorphism were not allowed.

Between one and three pairs of primers for each selected locus were synthesized by Eurofins Genomics (Huntsville, Alabama, USA). To assess the primer performance, we tested the amplifications in a set of *T. hemsleyanum* individuals (see Additional file 5: Table S3) representing all of the chloroplast haplotypes and populations reported in the previous study [6] via PCR. Our PCR recipe (25-μL reactions) was as follows: 5 μL of 5×Buffer (Mg^+2^ free), 1.25 μL MgCl_2_ (50 mM), 2 μL dNTP (5 mM), 1.25 μL of each primer (10 μM), 0.25 μL (5 units/μL) of Taq polymerase, and 60 ng template DNA (1.5 μL). We adopted the following PCR cycling conditions in a Biometra T3 Thermocycler (Whatman Biometra, Goettingen, Germany): a denaturing step at 94°C for 5 min, followed by 35 cycles of 30 s at 94°C, annealing at a specific temperature (optimized *T*_m_ for each locus presented in Additional file 5: Table S1) for 30 s and extension for 60 s, and a final extension for 10 min at 72°C.

For primer pairs that consistently yielded a single band in at least 90% of the individuals tested, we performed bidirectional sequencing for the PCR products at the University of Florida Interdisciplinary Center for Biotechnology Research. When more than one band amplified, we isolated bands, reamplified, and sequenced directly. Individual alleles (haplotypes) were determined from diploid nuclear loci using the software phase v2.1.1 [72], considering a threshold of 60% (p = q = 0.6) to reduce the number of genotype uncertainties with little or no increase in false positives [73]. The input files for software phase were created in seqphase [74]. If cloning was necessary, PCR products were purified, ligated into PMD19-T vector (Takara), and transformed into DHB-5α–competent cells (Invitrogen, Carlsbad, California, USA), reamplified, and sequenced. All of the generated sequences were edited, assembled, and aligned in geneious v7.1.7 [75].

We estimated the numbers of polymorphic sites, numbers of haplotypes, levels of nuclear sequence diversity (*π*_T_) for these SCNGs, and carried out tests of departure from the neutral model based on Tajima’s *D* [76] and Fu & Li’s D* and F* statistics [77] using dnasp v5.1 [78]. Genealogical relationships of the haplotypes identified at each nuclear locus were constructed from a 95% statistical parsimony network using tcs v1.21 [79]. To infer a more robust divergence and demographic history of *T. hemsleyanum*, we used the ‘isolation with migration’ model (IM) [80, 81] as implemented in ima2 [82] to estimate population rate parameters (*Θ*) and effective population sizes (*N*_e_) of the *SW* lineage in Southwest China (*Θ*_SW_), the *CSE* lineage in Central-South-East China (*Θ*_CSE_), and their common ancestral population (*Θ*_A_; *Θ* = 4*N*_e_*u*), as well as bidirectional migration rates (*m*_SW–CSE_ and *m*_CSE–SW_; *M* = *m*/*u*) and divergence times (*τ* = *tu*) between the two lineages. All parameters in the IM model are scaled by the neutral mutation rate (*u*). In our coalescence analyses, we jointly employed the eight nuclear loci identified here and the previously obtained sequences of three cpDNA regions (*petL–psbE*, *trnK–matK*, *rbcL*), because the cpDNA genealogical patterns [6] were mostly congruent with those retrieved from the nuclear loci (see Results). IM model involves several simplifying assumptions, e.g. no recombination within loci, free recombination among loci, no population structure within each species or populations, no genetic contribution from unsampled populations or species, and selective neutrality [80, 81]. However, recent simulation studies have revealed that ima parameter estimates are robust to small to moderate violations of IM model assumptions, and to significant levels of recombination when data sets are pared down to apparently nonrecombining blocks [83]. In this study, we determined the longest non-recombining block for each locus using the program imgc [84].

We applied the infinite sites (IS) model of nucleotide substitution to nuclear loci and the Hasegawa, Kishino and Yano (HKY) model to chloroplast loci as suggested in the ima2 software manual ([80]; https://bio.cst.temple.edu/~hey/software). We used locus-specific inheritance scalars to account for autosomal (nuclear loci = 1) and maternal (chloroplast loci = 0.5) inheritance. Based on the estimated divergence time and the average divergence of sequences between *SW* and *CSE* lineages (*c*. 5.07 Ma) [6], the substitution rate (substitution /site/year) for each locus was calculated as between 10^-10^ and 10^-9^ s/s/y. For the combined cpDNA sequences, an average substitution rate of 5×10^-10^ s/s/y, as estimated from our previous clock-calibrated beast tree of *Tetrastigma*, was adopted [74].

The geometric average mutation rate of the two marker sets was used to rescale the ima parameter estimates from the combined analysis. After deleting the first 10^5^ generations as burn-in, five independent Markov Chain Monte Carlo (MCMC) runs of 10 million generations were conducted under a geometric heating scheme (*h*_n_ = 40; *h*_a_ = 0.979, *h*_b_ = 0.60), and trees were sampled every 100 steps. The convergence of parameter estimates was assessed by monitoring the effective sample size (ESS > 300), the swapping rates between successive chains of MCMC, and the trend-line plots of the parameters.

## Additional files

Additional file 1: **Figure S1.** Size frequency distribution of contigs (a, *CSE* lineage; b, *SW* lineage) and unigenes (c, *CSE* lineage; d, *SW* lineage). (XLS 1093 kb)

Additional file 2: **Figure S2.** Distribution of gene ontology (GO) classifications for the 3737 orthologous pairs with *K*_*a*_/*K*_*s*_ ratios significantly < 0.5. The y-axis indicates the percent (left y-axis) or number of unigenes (right y-axis) per (sub-) category. (PDF 436 kb)

Additional file 3: **Figure S3.** Frequency distribution of EST-SSR unit size in the transcriptomes of *CSE* and *SW* lineages. (PDF 348 kb)

Additional file 4: **Figure S4.** Frequency distribution of EST-SSR repeat motifs (mono- to tri-nucleotide motifs) between *CSE* and *SW* lineages. (PDF 337 kb)

Additional file 5: **Table S1.** Characteristics of the twelve nuclear primer pairs newly developed in this study based on SCNG loci identified by different approaches. **Table S2.** Full list of 1018 SCNG loci identified from the transcriptomes of CSE and SW lineage, with their basic information including identification approaches, substitution rates between lineages and gene annotations. '+' denotes the approaches that supported the single-copy status of the locus. **Table S3.** List of individuals included in this study, with corresponding sampling localities and GenBank accession numbers. (PDF 415 kb)

Additional file 6: **Figure S5.** The pairwise identity of each SCNG pair between the two lineages. Each dot in the scatter diagram denotes a SCNG locus, and the blue colored ones represent those genes under purifying selection). (PDF 177 kb)

Additional file 7: **Figure S6.** Agarose-gel electrophoresis patterns of a subset of SCNG primer pairs for *T. hemsleyanum (PDF 148 kb)*

Additional file 8: **Figure S7.** TCS-derived network of genealogical relationships among the identified haplotypes for each of the SCNG locus (PDF 198 kb)

## Abbreviations

APVO: *Arabidopsis*-*Populus*-*Vitis*-*Oryza*
BGI: Beijing Genome Institute
CDS: coding region sequences
COG: Cluster of Orthologous Groups
cpDNA: Chloroplast DNA
*CSE*: Central-South-East
EST-SSR: Expressed sequence tag–simple sequence repeat
GO: Gene ontology
HKY model: Kishino and Yano model
HPD: Highest probability density
IM: Isolation with migration
*K*_*a*_: Nonsynonymous
KEGG: Kyoto Encyclopedia of Genes and Genomes
*K*_*s*_: Synonymous
MCMC: Markov Chain Monte Carlo
MISA: MIcroSAtellite
MLEs: maximum-likelihood estimates
MPP: marginal posterior probability
NCBI: National Center for Biotechnology Information
NGS: Next-generation sequencing
Nr: Non-redundant
RNA-Seq: Sequencing of RNA
SCNGs: Southwest (*SW*) single-copy nuclear genes
SRA: Sequence Read Archive
WTE: Warm-temperate evergreen
