# Supplementary figures and images for: Evolutionary insights from comparative transcriptome and transcriptome-wide coalescence analyses in Tetrastigma hemsleyanum
Source: BMC Plant Biol. 2018 Sep 24;18:208. doi: 10.1186/s12870-018-1429-8 (PMC6154912; doi:10.1186/s12870-018-1429-8)

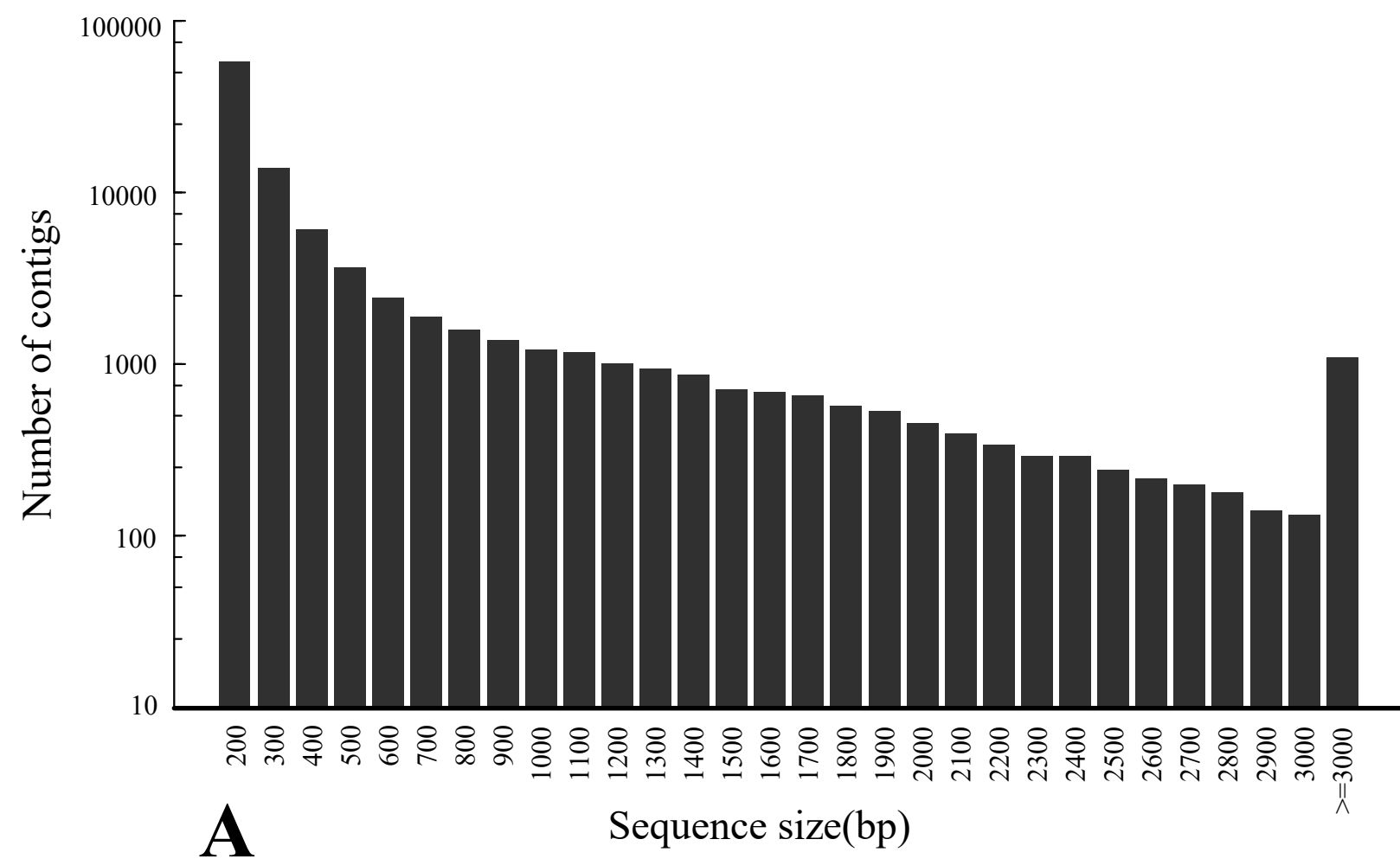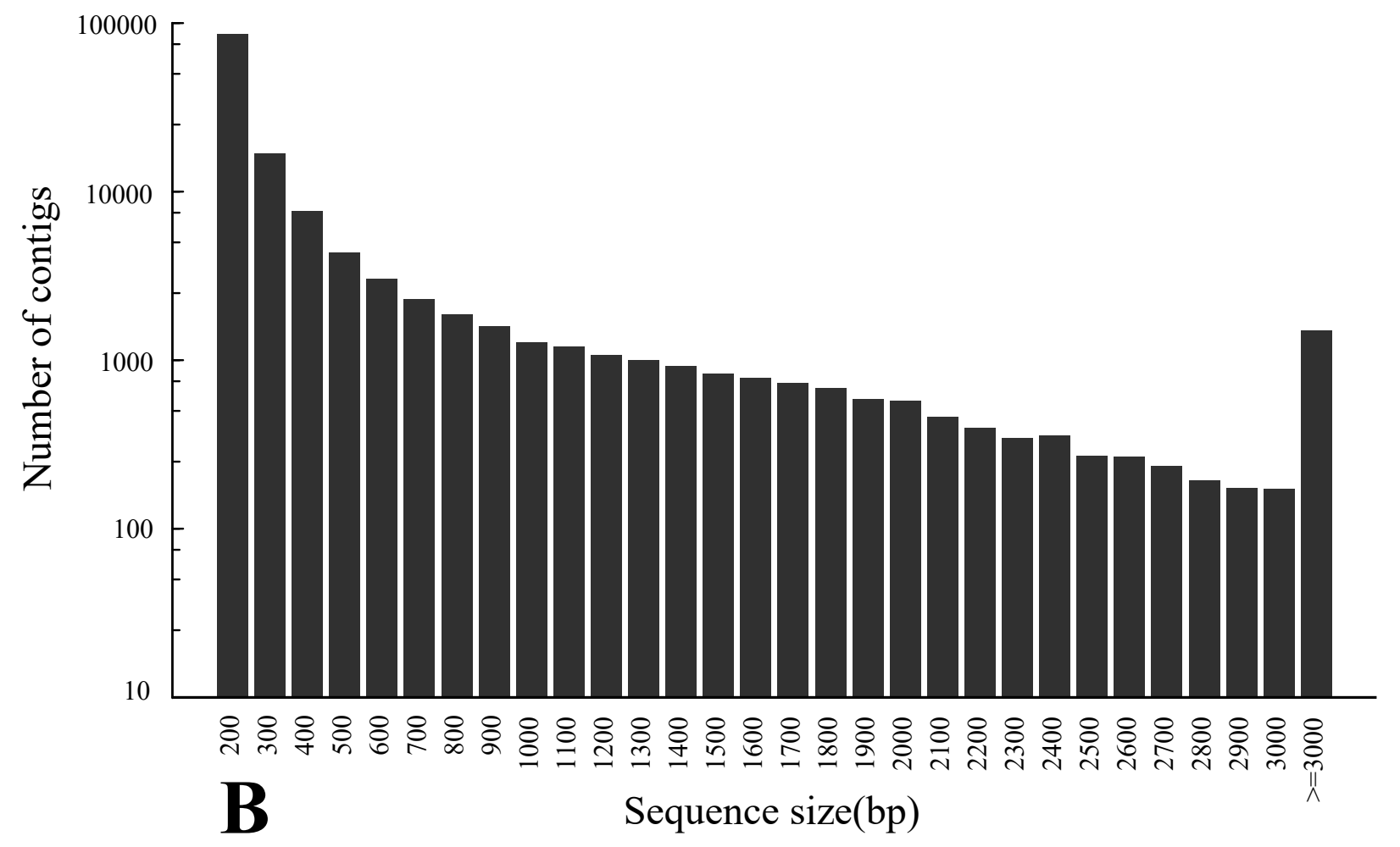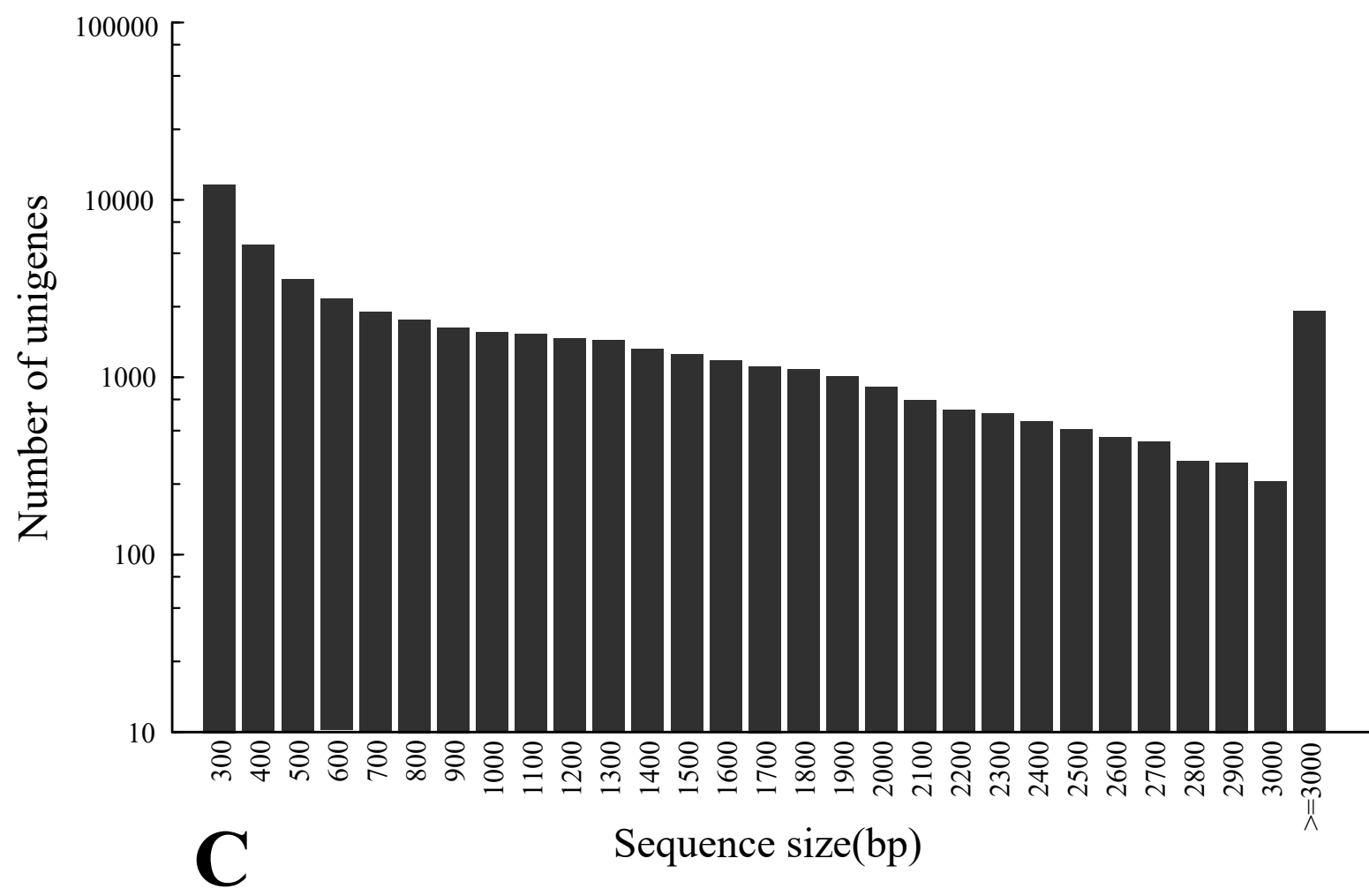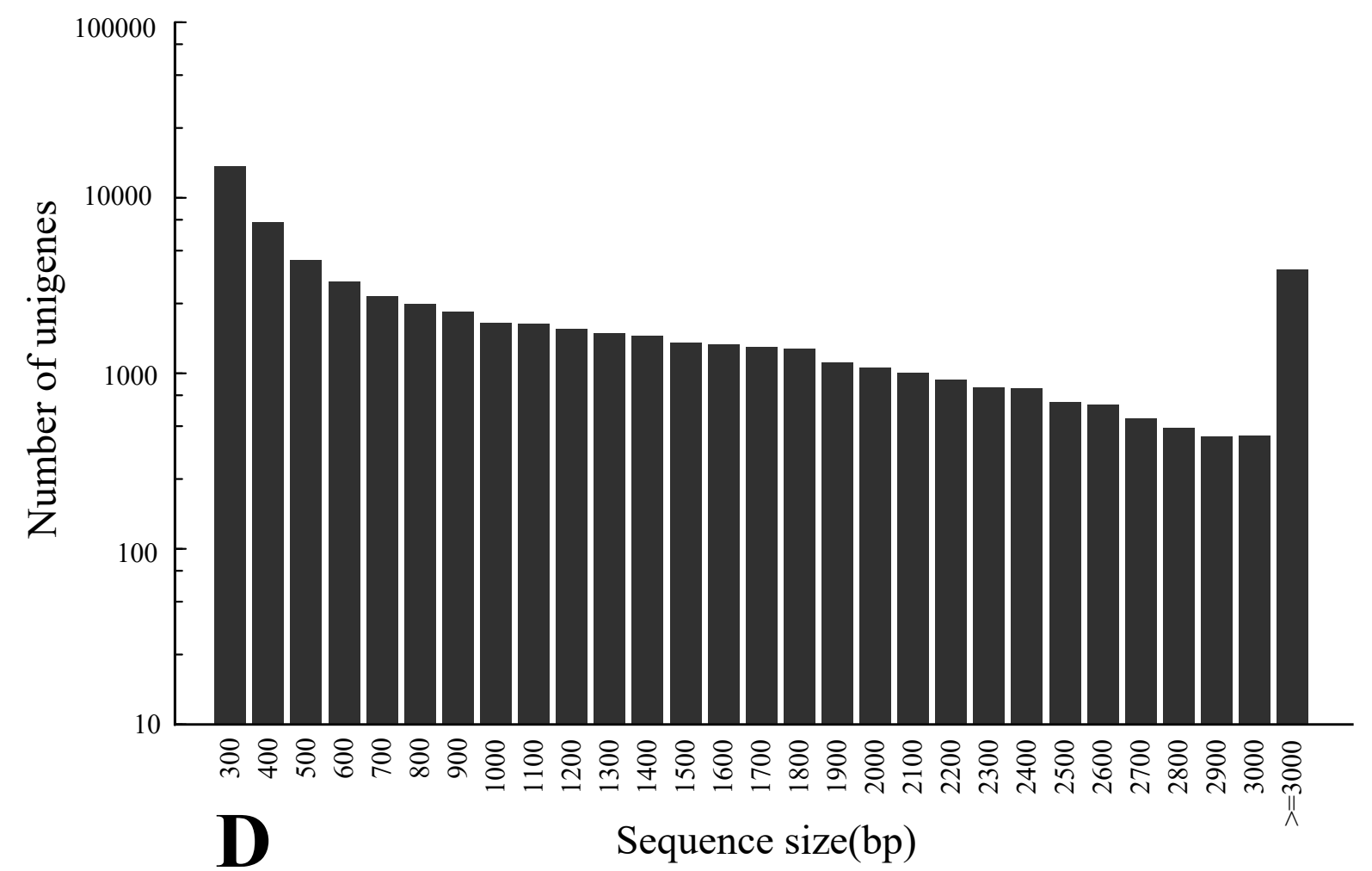

Supplement: Supplementary file 2 — Figure S2. Distribution of gene ontology (GO) classifications for the 3737 orthologous pairs with Ka/Ks ratios significantly < 0.5. The y-axis indicates the percent (left y-axis) or number of unigenes (right y-axis) per (sub-) category. (PDF 436 kb) [file 12870_2018_1429_MOESM2_ESM.pdf]

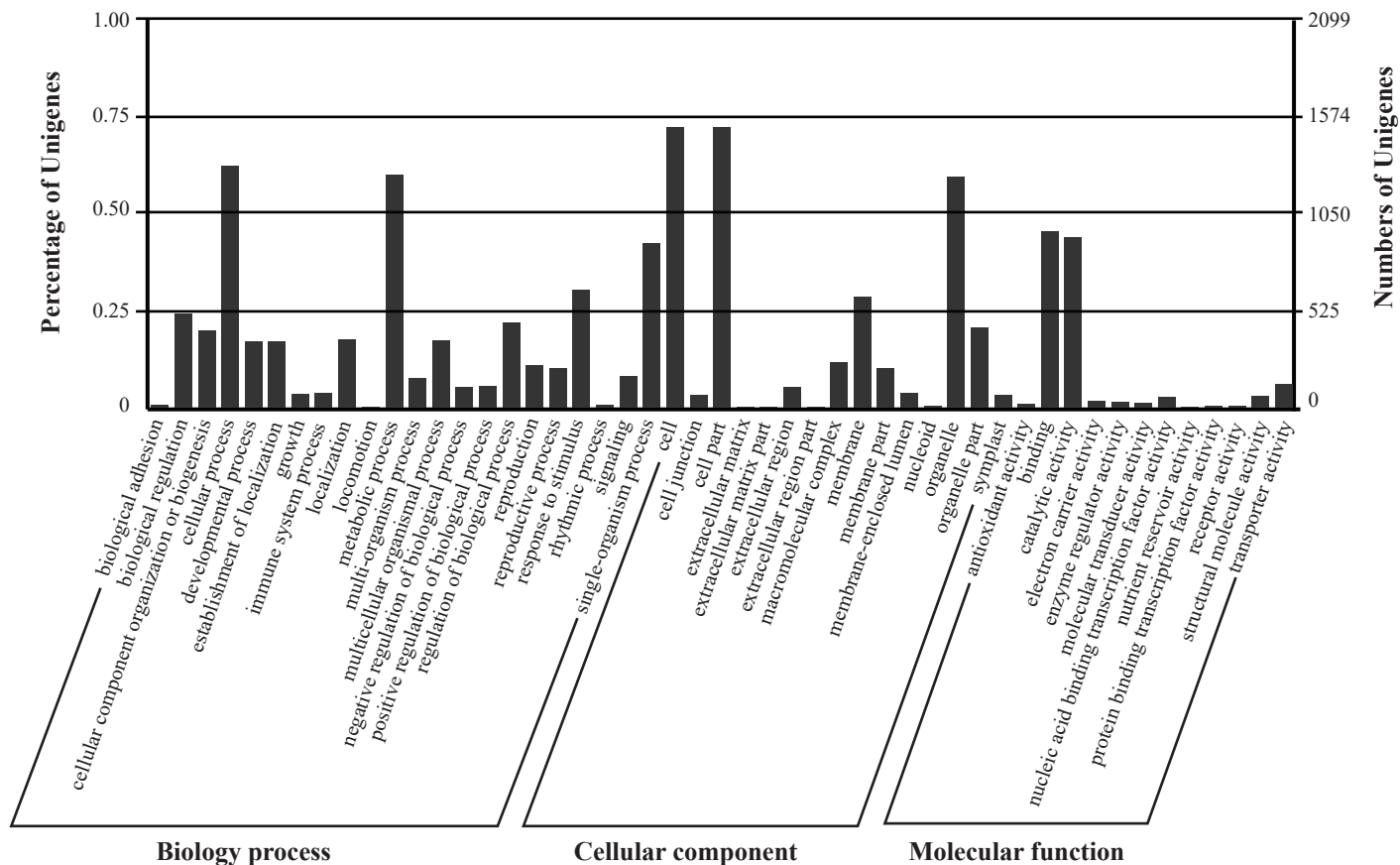

Supplement: Supplementary file 3 — Figure S3. Frequency distribution of EST-SSR unit size in the transcriptomes of CSE and SW lineages. (PDF 348 kb) [file 12870_2018_1429_MOESM3_ESM.pdf]

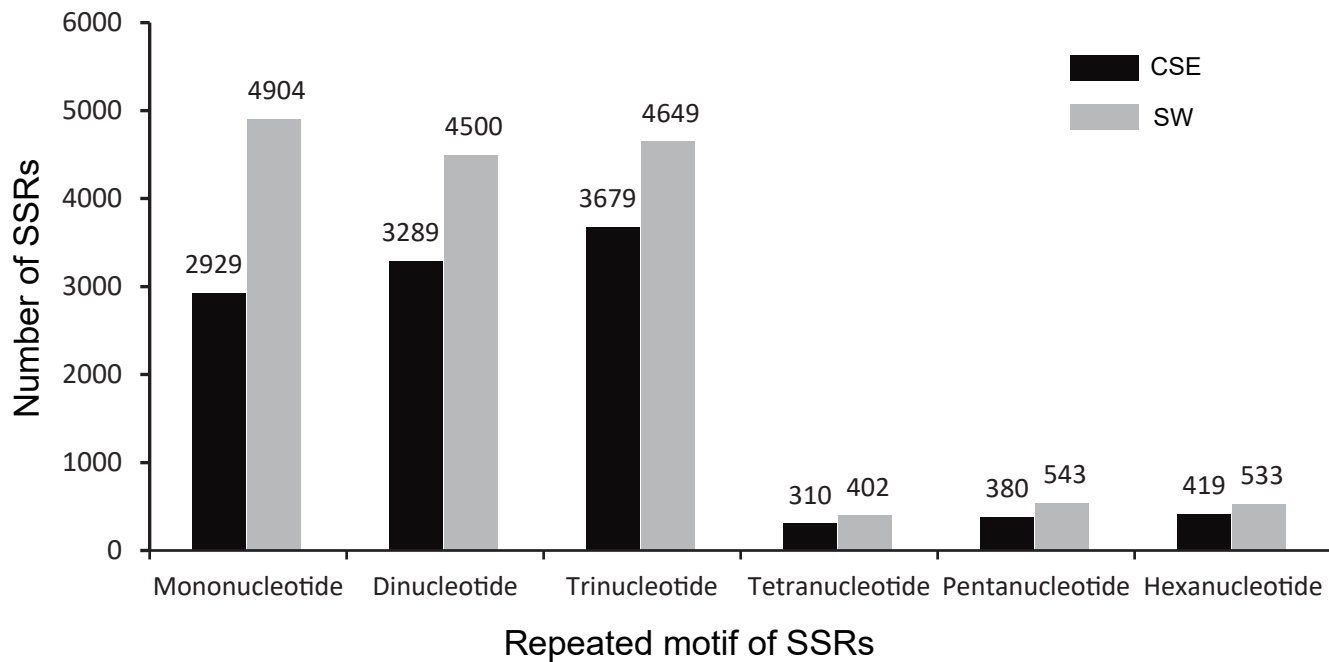

Supplement: Supplementary file 4 — Figure S4. Frequency distribution of EST-SSR repeat motifs (mono- to tri-nucleotide motifs) between CSE and SW lineages. (PDF 337 kb) [file 12870_2018_1429_MOESM4_ESM.pdf]

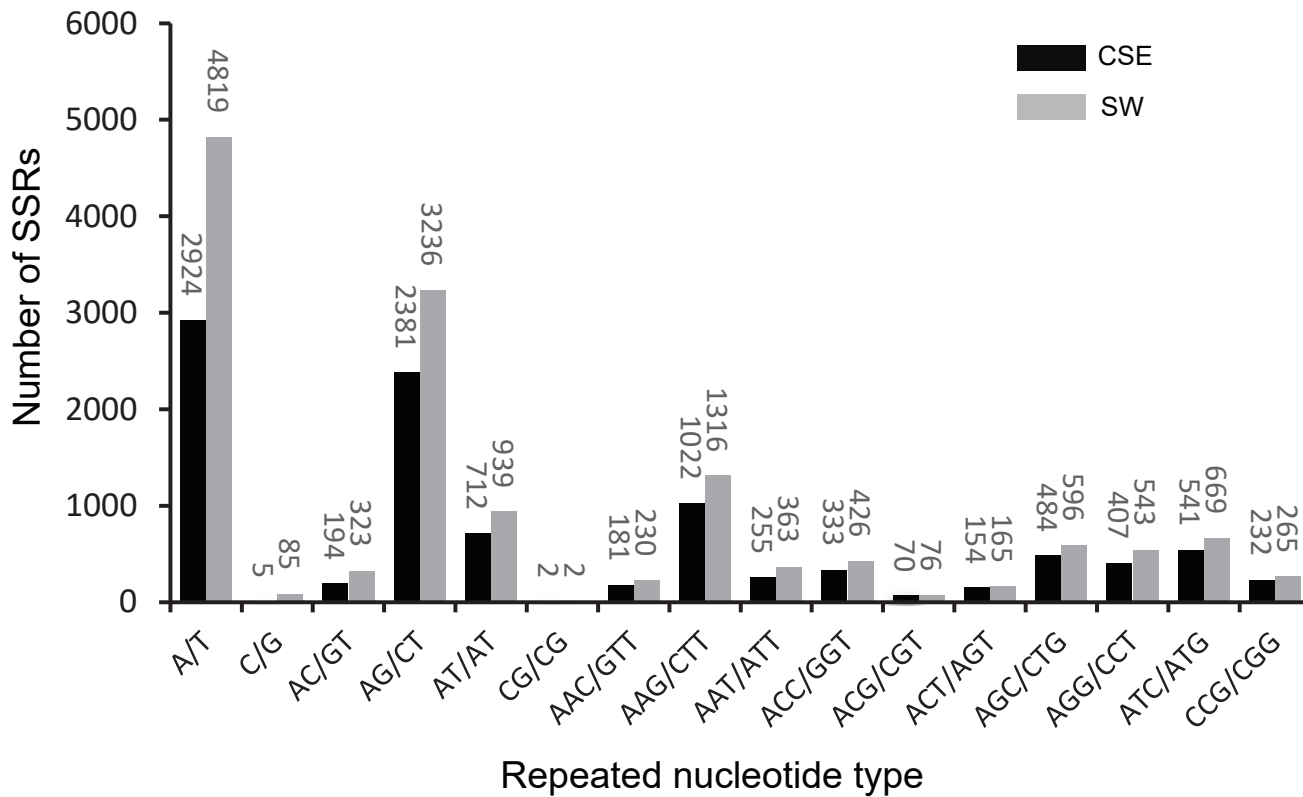

Supplement: Supplementary file 5 — Table S1. Characteristics of the twelve nuclear primer pairs newly developed in this study based on SCNG loci identified by different approaches. Table S2. Full list of 1018 SCNG loci identified from the transcriptomes of CSE and SW lineage, with their basic information including identification approaches, substitution rates between lineages and gene annotations. '+' denotes the approaches that supported the single-copy status of the locus. Table S3. List of individuals included in this study, with corresponding sampling localities and GenBank accession numbers. (PDF 415 kb) [file 12870_2018_1429_MOESM5_ESM.pdf]

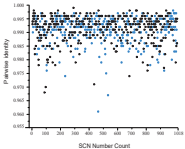

Supplement: Supplementary file 6 — Figure S5. The pairwise identity of each SCNG pair between the two lineages. Each dot in the scatter diagram denotes a SCNG locus, and the blue colored ones represent those genes under purifying selection). (PDF 177 kb) [file 12870_2018_1429_MOESM6_ESM.pdf]

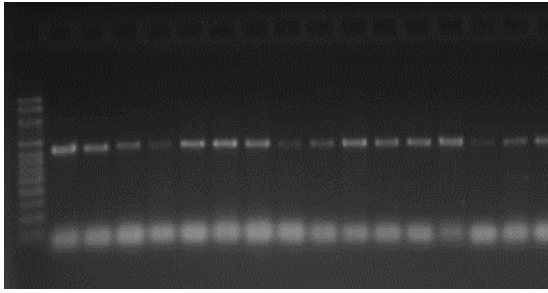

ThR-3

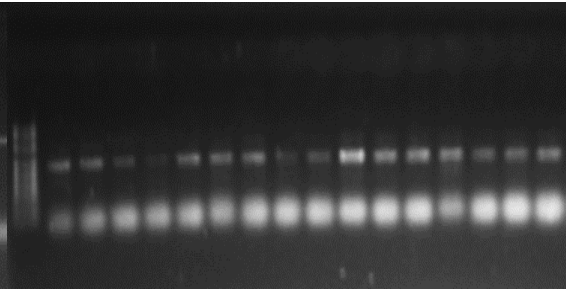

ThR-6

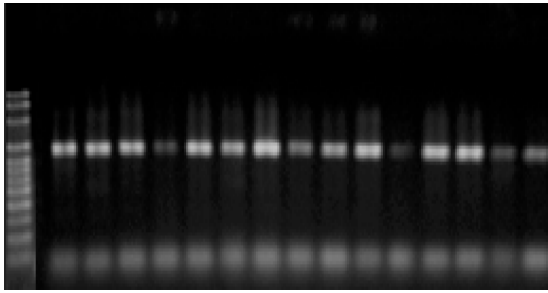

ThR-7

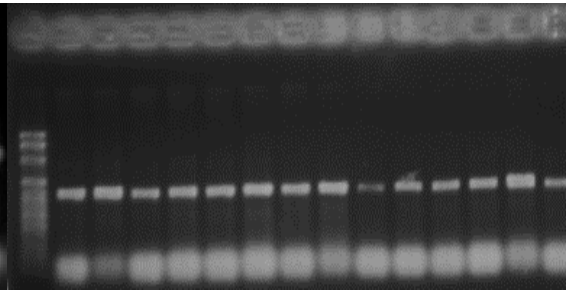

ThR-28

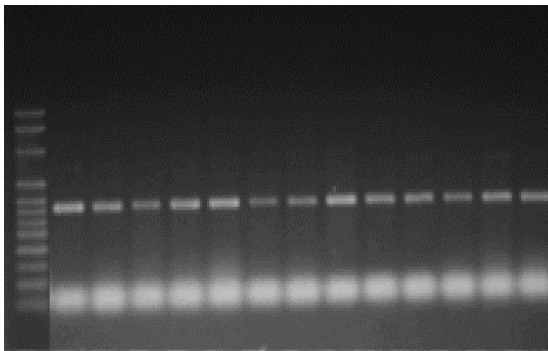

Th-41

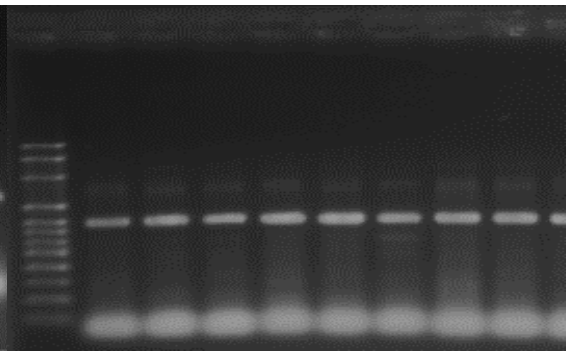

ThR-11

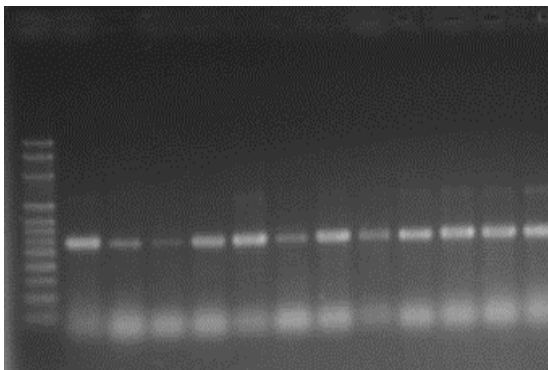

ThR-31

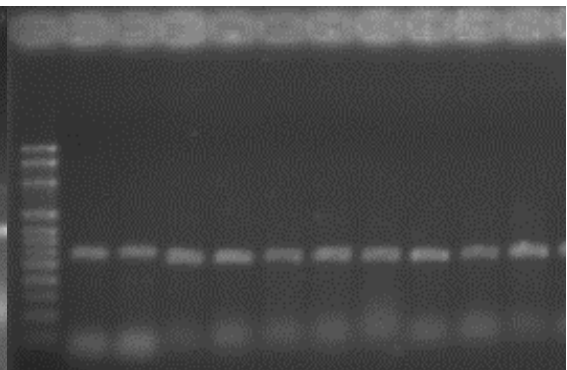

ThR-34

Supplement: Supplementary file 7 — Figure S6. Agarose-gel electrophoresis patterns of a subset of SCNG primer pairs for T. hemsleyanum (PDF 148 kb) [file 12870_2018_1429_MOESM7_ESM.pdf]

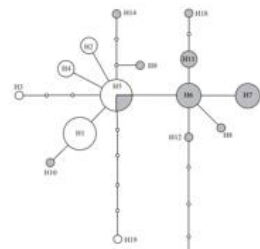

Th41

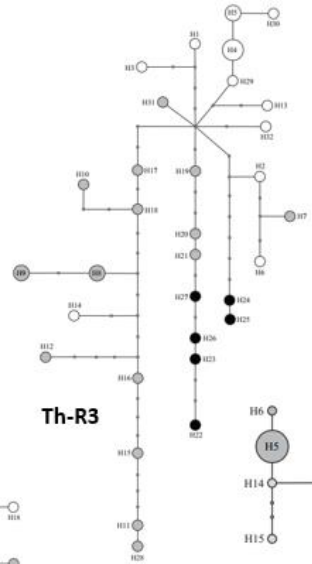

Th-R3

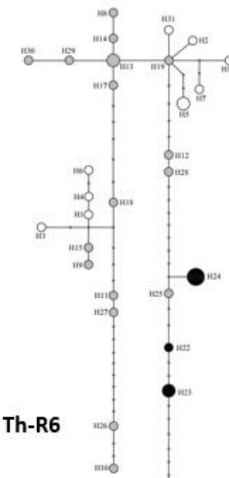

Th-R6

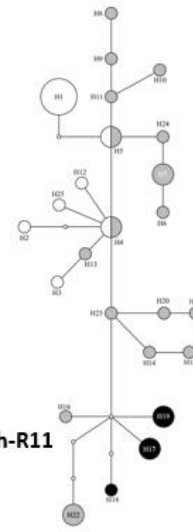

Th-R11

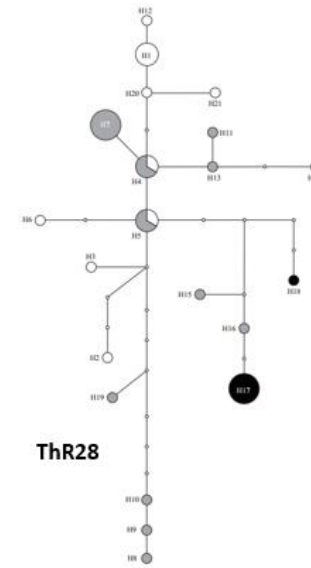

ThR28

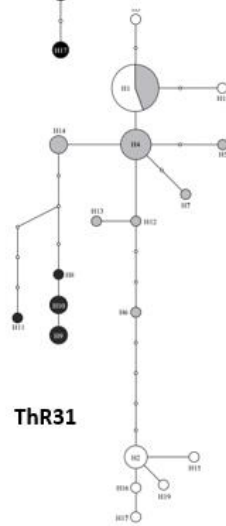

ThR31

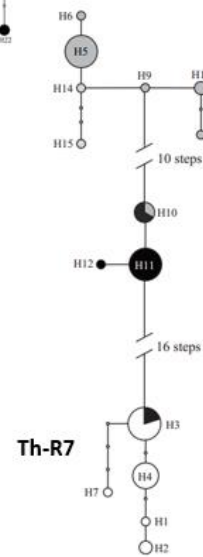

Th-R7

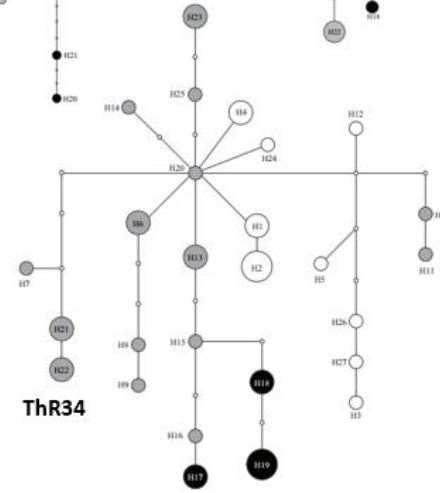

ThR34

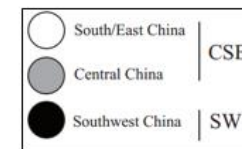

Supplement: Supplementary file 8 — Figure S7. TCS-derived network of genealogical relationships among the identified haplotypes for each of the SCNG locus (PDF 198 kb) [file 12870_2018_1429_MOESM8_ESM.pdf]
